# Flexibility along the Neck of the Neogene Terror Bird *Andalgalornis steulleti* (Aves Phorusrhacidae)

**DOI:** 10.1371/journal.pone.0037701

**Published:** 2012-05-25

**Authors:** Claudia P. Tambussi, Ricardo de Mendoza, Federico J. Degrange, Mariana B. Picasso

**Affiliations:** 1 División Paleontología Vertebrados, Museo de La Plata, Paseo del Bosque s/n, La Plata, Argentina; 2 Consejo Nacional de Investigaciones Científicas y Técnicas CONICET, Ciudad Autónoma de Buenos Aires, Argentina; 3 Centro de Investigaciones en Ciencias de la Tierra CICTERRA, Córdoba, Argentina; Monash University, Australia

## Abstract

**Background:**

*Andalgalornis steulleti* from the upper Miocene–lower Pliocene (≈6 million years ago) of Argentina is a medium-sized patagornithine phorusrhacid. It was a member of the predominantly South American radiation of ‘terror birds’ (Phorusrhacidae) that were apex predators throughout much of the Cenozoic. A previous biomechanical study suggests that the skull would be prepared to make sudden movements in the sagittal plane to subdue prey.

**Methodology/Principal Findings:**

We analyze the flexion patterns of the neck of *Andalgalornis* based on the neck vertebrae morphology and biometrics. The transitional cervical vertebrae 5th and 9th clearly separate regions 1–2 and 2–3 respectively. Bifurcate neural spines are developed in the cervical vertebrae 7th to 12th suggesting the presence of a very intricate ligamentary system and of a very well developed epaxial musculature. The presence of the *lig. elasticum interespinale* is inferred. High neural spines of R3 suggest that this region concentrates the major stresses during downstrokes.

**Conclusions/Significance:**

The musculoskeletal system of *Andalgalornis* seems to be prepared (1) to support a particularly big head during normal stance, and (2) to help the neck (and the head) rising after the maximum ventroflexion during a strike. The study herein is the first interpretation of the potential performance of the neck of *Andalgalornis* in its entirety and we considered this an important starting point to understand and reconstruct the flexion pattern of other phorusrhacids from which the neck is unknown.

## Introduction

Neck length and neck posture are both relevant to give the appropriate position of the head during all kinds of behaviors [1], [2]. For activities such as mating or defense the neck has the ability to perform more complex movements [3] linked to the cervical morphology. The head centre of gravity over the feet is deeply correlated with the erect posture on land and also depends on the movements of the neck. In normal stance birds (and all amniotes) maintain vertical its cervical column [4], [5].

**Figure 1:**
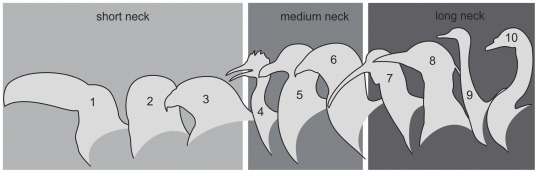
Comparison between external neck guises in selected birds. 1 toucan, 2 owl, 3 eagle, 4 tinamous, 5 seagull, 6 seriema, 7 ibis, 8 egret, 9 greater rhea, 10 swan.

Size, morphology and number of vertebrae are three important variables involved in the motion of the neck [6], [7], [8]. Birds have a highly variable number of cervical vertebrae (9–11 in some parrots, 23–25 in swans) but, whatever the number, their necks look shorter or longer depending on the neck-folding (Fig. 1). All birds have a flexible “S” shaped neck but the flexion capacity varies between them and for sure, the arrangement is a consequence of an intricate system made by muscles, ligaments and complex articulations. As flexion increases, the distance between the head and the thorax will be shorter and, under these circumstances the external profile of the animal’s neck region shortens [9].

**Figure 2:**
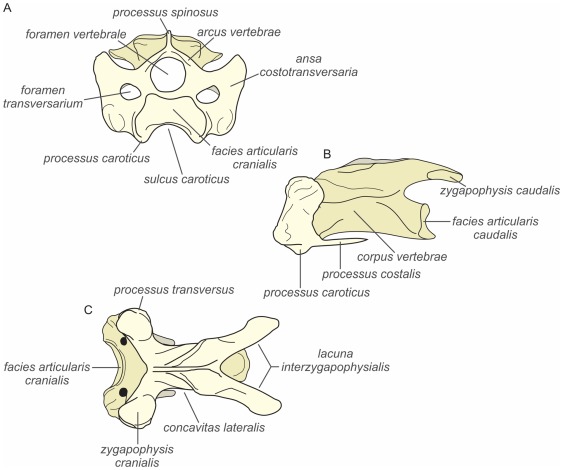
Features of the 5th cervical vertebrae in the seagull *Larus* sp. A, cranial view; B, lateral view; C, dorsal view.

Each vertebra articulates with the adjacent through a saddle-shaped region (*articulatio intercorporalis* in the sense of [10]) located at the base of the vertebral body (*corpus vertebrae*) (Fig. 2) and two additional sliding surfaces located in the top (*articulatio zygapophysialis cranialis* and *caudalis*) [8]. This morphology allows large dorsoventral movements of the neck but prevents rotation, which is generally small.

Boas [6] divided the cervical column of birds in three main regions according to the dorsoventral bending: the most rostral is the region 1 in which ventral flexion is prevalent, region 2 in which dorsal flexion prevails and region 3 in which both dorsal and ventral flexion are limited. The boundaries between these regions are given by transitional vertebrae that have a particular morphology. Knowing the vertebral morphology of each region one can infer what kind of movements could be possible.

*Andalgalornis steulleti* (Kraglievich 1931), from the upper Miocene–lower Pliocene (≈6 million years ago) of Argentina, was a medium-sized patagornithine phorusrhacid of about 40 kg body mass, 1.4 m height, and 370 mm total skull length [11], [12]. Together with the long legs, the atypical large skull with high and narrow beak is a characteristic feature of all phorusrhacids [13]. The Phorusrhacidae are often regarded as apex predators of the South American Tertiary environments sharing this role with large now-extinct marsupials [14]. Popularly known as “terror birds” (a name applied at least to the giant forms) there are no close modern analogs which facilitate interpretations about their biology.

Here we analyze the flexion patterns of the neck of *Andalgalornis* based on the neck vertebrae morphology and biometrics. Complete columns among phorusrhacids remains are scare and in most cases in those specimens where the column is quite complete, the vertebrae are severely damaged. So far, the column of *Andalgalornis* is the most complete and well preserved known of a terror bird. The study here is the first interpretation of the potential performance of the neck of *Andalgalornis* in its entirety and we considered this an important starting point to understand and reconstruct the entire neck of other phorusrhacids from which the neck is unknown or severely damaged.

The vertebral morphology of *Andalgalornis* has not been extensively described in the literature. In their original description of the only complete column known for the species, Patterson and Kraglievich [15] recognized the presence of 17 presynsacral vertebrae (11 cervicals, 2 cervicodorsals and 4 dorsals) of which 13 form the neck. Their description is limited to the size comparison of the vertebral body and only in a few vertebrae did they also compare the height of the neural spine.

We will dwell on the comparison between several extant birds of different size and habits in order to establish a correlation between cervical morphology and mode of life. Such anatomical analysis is not available for any phorusrhacid, while it is a first requirement for any quantitative analysis in comparative, functional or ecological morphology.

## Results

### Anatomical Description

**Figure 3:**
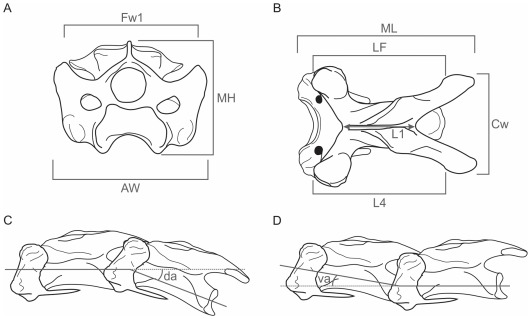
Schematic vertebrae showing the measurements and angles taken. A, cranial view; B, dorsal view; C–D, lateral view. ML maximum length, MH maximum height, LF in dorsal view, distance between the *facies articularis craniali*s and *caudalis*, L1 in dorsal view, distance between the cranial and posterior borders of the *foramen vertebrale*, L4 in ventral view, maximum length of the *corpus*, AW cranial width, Fw1 distance between both *zygapophysis cranialis*, Cw caudal width. L1, L4 and Fw1 are following Guinard *et al.* (2009: Fig. 2), I1 development and cranial projection of *facies articularis cranialis* (I1 =  L4-L1/L4), I2 elongation index (I2 = L4/Fw1), the later in the same way as Guinard *et al.* (2009), da dorsal angle, va ventral angle, c. circa because deformation.

We use the term cervical vertebrae to mean the true cervical (without ribs or with fused ribs) and the cervicodorsal vertebrae to refer to vertebrae with free ribs that do not contact with the sternum [16]. Skeletal characters used in the descriptions are shown in Figure 2 while Figure 3 exhibits the measurements and angles analyzed in this paper. Our treatment here follows the concept of regions defined by types of mobility, which are separated by transitional vertebrae: fifth cervical vertebra and ninth cervical vertebra in *Andalgalornis*. Comparisons [between brackets] with other phorusrhacids were made when possible.

### Region 1 (R1)

**Figure 4:**
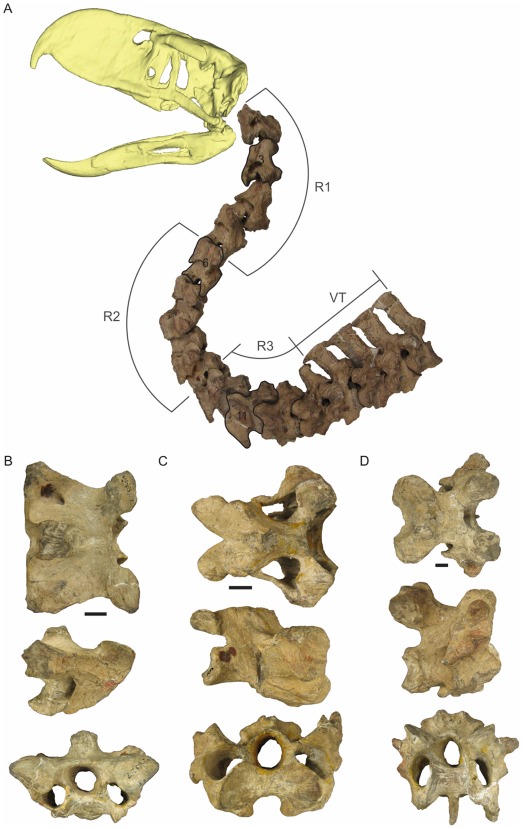
Complete neck of *Andalgalornis steulleti* FMNH - P14357 and selected vertebrae. A, neck with R2 in maximum dorsal flexion. R1 region 1, R2 region 2, R3 region 3, VT cervico dorsal vertebrae; B, vertebra 3 (R1); C, vertebra 6 (R2); D, vertebra 11 (R3). Top line, dorsal view; Midline, right lateral view; Bottom line, cranial view. Scale 1 cm.

This region includes cervical vertebrae one to five. In general, they are robust and short (I2 with lower values than those from the R2), with weakly developed *facies articularis cranialis* (I1 with smaller values than R2), and the *facies articularis caudalis* exposed when viewed dorsally (Fig. 3, 4). This is a more massive (cohesive) region of the neck that is capable of downward (ventral) flexion but only slightly upward (dorsal) flexion is possible (see above and Table 1).

**Table 1:** Angles between vertebrae taken with goniometer.

Region	Vertebra	Ventral angle	Dorsal angle
R1	V3–V4	–	–
	V4–V5	30°	20°
R2	V5–V6	18°	27°
	V6–V7	11°	40°
	V7–V8	9°	43°
	V8–V9	4°	45
R3	V9–V10	0°	36°
	V10–V11	10°	30°
	V11–V12	5°	27°
	V12–V13	0°	10°

– Not measured.

The atlas (first vertebra) of *Andalgalornis* is unknown.

The second cervical vertebra, the axis, is badly deformed mainly in the corpus. It appears to be long and ventrally rough with three ridges, one central and two laterally placed [in *Procariama simplex* Rovereto 1914 the corpus is smooth, and there is only one central ridge]. The *zygapophyses cranialis* are small. Viewed laterally, behind the *zygapophysis,* the *ansa costotransversaria* is located [absent in *P. simplex*] with small *processus costalis*. The *zygapophyses caudalis* are large, and ventrally concave. The *facies articularis caudalis* is dorsocaudally oblique [strongly dorsocranially oblique in *Psilopterus* Moreno and Mercerat 1891 *sensu* [17]. The *processus spinosus* is high, thick and has a wide posterior fossa for ligament insertion.

The *corpus* of cervical vertebra 3 is narrow and ventrally convex [flat in *P. simplex*]. The *facies articularis cranialis* is thin viewed cranially [broad in *P. simplex*] and extensive in the ventral region. The *zygapophyses cranialis* face craniodorsally and are slightly convex. The *ansa costotransversaria* is robust and craniocaudally disposed, the *tuberculum ansae* is thick but slightly marked [absent in *P. simplex*]. The *processus costalis* is small. The *facies articularis caudalis* is proportionately smaller and widely dorsally directed. In dorsal view, the general shape of the vertebra is trapezoidal, wider than longer, with the *concavitas lateralis* hidden. The *processus spinosus* is high and wide, caudally located, with an elliptic deep fossa placed caudally which possibly corresponds to the insertion of the *ligamentum elasticum interlaminare.* The high *tori dorsalis* are laterocaudally located above the most lateral sector of the *zygapophysis caudalis*. The latter is large and slightly concave.

Cervical vertebra 4 presents the *corpus* proportionately larger, with a ventrally-directed crest (*processus ventralis corporis*) descending from the midline of the *centrum* [crest absent in *Procariama*]. The *processi caroticus* are low, slender and extended ventrally around the *facies articularis cranialis.* The *facies articularis caudalis* is vertical [oblique in *Psilopterus*]. Viewed dorsally, the vertebra is trapezoidal. The *processus spinosus* is high and narrow and is located on the caudal portion of the vertebra. A posterior and very deep fossa is presented on the spine but narrower than that of the vertebra 3. Caudally, there is a small notch in the bar that connects both *zygapophysis caudalis*. The *tori dorsalis* are slightly proximal and lateralized and are higher than previous vertebrae.

### Transitional Vertebra between R1–R2 (Fig. 5)

The fifth cervical vertebra of *Andalgalornis* is the last of the first region [identical condition in *P. simplex* and *Mesembriornis milneedwardsi* Moreno 1889] presenting a transitional morphology [18]. The vertebral body is longer and broader [narrower in *Procariama*] than in the preceding vertebrae widening caudally. The elongation index is greater than that of the cranial vertebrae but lower than that of the vertebrae of R2 (Table 2). Ventrally, the *corpus* is slightly concave. The *facies articularis cranialis* disposes more vertically in relation to the cranial vertebrae (I1 greater than that of vertebrae 3 and 4). The *facies articularis caudalis* is vertically disposed. The *zygapophyses cranialis* are slightly vertical, with a slope directed craniomedially. Caudolaterally to the *facies articularis cranialis* are the well *processi transversus*. Baumel and Witmer [10] pointed out that cervical vertebrae have *processus transversus* indistinguishable from the *ansa costotransversaria* in mature birds. Skull morphology suggests that *Andalgalornis steulleti* FM-P14357 is an adult specimen and could even be of advanced maturity. The condition of having the *processi transversus* distinguishable of the *ansae costotransversaria* is indeed a feature rare among mature birds [10]. The *ansa costotransversaria* is robust and long craniocaudally, with a *tuberculum ansae* with low development [absent in *Procariama*]. Dorsally it is possible to observe the *concavitas lateralis* due to the reduction of the *lamina lateralis arcus* (that connects *zygapophysis cranialis* and *zygapophysis caudalis*). The *processus spinosus* is centrally placed and is smaller than in the preceding vertebrae. The caudal *lacuna interzygapophysialis* is more pronounced than in preceding vertebrae. It presents a “V” shape with its apex located at the same level than the small *tori dorsalis* [absent in *Procariama*], and ends in the most caudal section of the *zygapophyses caudalis*. This shape allows the vertebral accommodation during the dorsal flexion. The *zygapophyses caudalis* are small and their facets face lateroventrally [similar condition in *M. milneedwardsi*].

**Figure 5:**
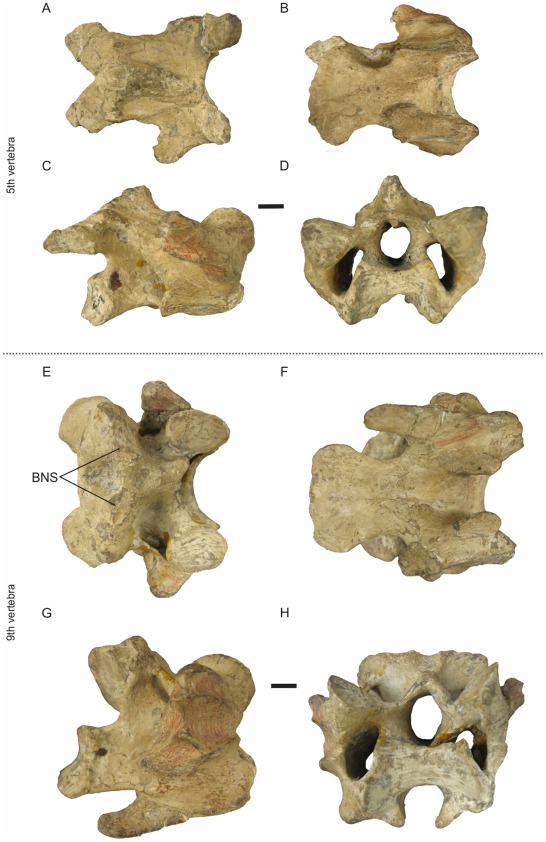
Transitional vertebrae of *Andalgalornis steulleti* FMNH - P14357. A, E dorsal view; B, F ventral view; C, G right lateral view; D, H cranial view. Scale 1 cm.

**Table 2:** Measurements and indices of 3^rd^ to 13^th^ *Andalgalornis steulleti* cervical vertebrae. Abbreviations in Figure 3.

	R1			R2				R3			
V	3	4	5	6	7	8	9	10	11	12	13
ML	68.92	69.13	78.92	79.64	72.19	69.63	69.28	69.70	72.55	67.23	78.83
MH	54.25	57.77	57.73	52.46	53.78	61.15	65.65	73.74	87.6	98.24	107.27
L1	44.82	45.35	46.49	40.06	40.51	31.63	34.45	32.56	36.87	39.25	45.94
LF	47.94	54.39	56.29	56.77	56.60	53.50	49.78	54.12	55.38	65.71	58.30
L4	54.42	52.47	56.40	59.78	60.63	59.45	58.96	59.20	57.61	51.30	45.91
AW	70.23	60.99	72.73	73.31	69.70	73.43	75.06	78.36	81.7	93.3	85.66
Fw1	70.26	59.82	59.55	c.57.11	c.50.15	c.51.83	54.86	59.60	56.97	62.85	50.14
Cw	75.84	69.4	47.27	43.94	42.33	52.52	56.39	56.62	52.85	52.25	43.7
I1	0.065	0.166	0.174	0.294	0.284	0.408	0.307	0.398	0.334	0.402	0.212
I2	0.774	0.887	0.947	1.046	1.208	1.014	1.074	0.993	1.011	0.816	0.915

### Region 2 (R2)

In *Andalgalornis steulleti* vertebrae of R2 do not exceed 35% of all neck vertebrae (Table 3), including vertebrae 6 to 9.

**Table 3:** Comparison of the number of vertebrae per region of *Andalgalornis* with selected taxons with and without bifurcated neural spines.

	R1	R2	R3	BNS	T
*Andalgalornis*	5v	4v	4v	5v	13
	38.46%	30.76%	30.76%	38.46%	
*Rhea*	5v	9v	3v	6v	17
	29.41%	52.94%	17.64%	35.29%	
*Chunga*	5v	6v	2v	absent	13
	38.46%	46.15%	15.38%	−	
*Theristicus*	5v	7v	5v	8	17
	29.41%	41.17%	29.41%	47.05%	
*Buteo*	5v	4v	5v	absent	14
	35.71%	28.57%	35.71%	−	
*Tyto*	5v	4v	4v	absent	13
	38.46%	30.76%	30.76%	−	
*Ramphastos*	5v	5v	4v	absent	14
	35.71%	35.71%	28.57%	−	
*Cygnus*	12v	9v	2v	absent	23
	52.17%	39.13%	8.69%	−	
*Nycticorax*	5v	8v	4v	absent	17
	29.41%	47.05%	23.53%	−	
*Rhynchotus*	5v	8v	3v	absent	16
	31.25%	50%	18.75%	−	
*Larus*	5v	5v	3v	absent	13
	38.46%	38.46%	23.08%	−	

BNS vertebrae with bifurcated neural spines, T total vertebrae of the neck, v vertebrae.

In the sixth cervical vertebra (the first of this region) dominates the dorsal flexion (between vertebrae 5 and 6, Table 1). The *facies articularis cranialis* (Fig. 4) is wider than the anterior vertebrae and is perpendicular to the longitudinal axis of the neck (L2 higher than previous vertebrae). The *zygapophyses cranialis* have a medial slope and are smaller than the ones of the more cranial vertebrae. The *processi transversus* are vertical and lateral to the *zygapophyses cranialis* [oblique in *Procariama*]. These *processi* are posteriorly connected with the base of the *tori dorsalis* by thin projections. These projections are present in cervical vertebrae 6, 7, 8 and 9 of *A. steulleti* [barely pronounced in vertebra 6 and highly developed in vertebrae 8, 9, 10 and 11 in *P. simplex*; vertebra 7 is broken at the level of the *processus* and it is not possible to establish the presence of this feature; well developed in vertebrae 6 to 9 in *M. milneedwardsi*; occur in vertebrae 7, 8, 9 and 10 in the Cariamidae *Chunga*]. The *ansa costotransversaria* is robust and has a strong development of the *tuberculum ansae* and the *crista laterales* (for the muscle *intertransversarii* attachment). The *processus caroticus* is well marked and ventromedially directed. The *facies articularis caudalis* is wide. Dorsally, the *processus spinosus* is disposed caudally, allowing the accommodation of the previous vertebra during dorsal flexion. The *lamina lateralis* is coincident with the vertebral body [not in *Procariama*] and the *concavitas lateralis* is widely developed. The *processus spinosus* is reduced, like the *tori dorsalis* that constitutes low prominences.

The cervical vertebrae 6, 7 and 8 are very similar in morphology (Fig. 4). However, the 7th is different in that the *ansa costotransversaria* presents a further development of the *tuberculum ansae* and the *crista lateralis*, the *processi caroticus* are more robust, the *zygapophyses cranialis* are more inclined medially, the *crista transverso-oblicua* link the much reduced *processus spinosus* with the *tori dorsalis* and the area of insertion of the *lig. elasticum interlaminare* is deeper, forming a furrow from the neural arch to the *lacuna interzygapophysialis*.

The elongation index of the 8th cervical vertebra is less than those of the 6th and 7th. This vertebra presents the *facies articularis caudalis* not well extended laterally, adopting a square like form, the *processus spinosus* is small and is bifurcated [same condition in *P. simplex*] (see above). The *zygapophysis caudalis* are greatly separated from each other, increasing the angle of the *lacuna interzygapophysialis*.

### Transitional Vertebra between R2–R3 (Fig. 5)

The cervical vertebra 9 also shares the same morphology as the cervical vertebrae of the R2, but is wider (see AW in Table 2). The *processus spinosus* is bifurcated, the space between both spines is broad and the entire structure is higher than in the cranial vertebrae [18]. The *processi costalis* are robust being much extended caudally than in vertebrae of R2, almost reaching the end of the vertebral corpus. These *processi* are present in all the vertebrae previously described, but they are broken or eroded and comparative development can not be established. The caudal *lacuna interzygapophysialis* is bounded by three sides, taking a trapezoid shape [triangular *P. simplex*], with the longest side between the caudal ends of the *zygapophysis caudalis*.

### Region 3 (R3)

This region includes vertebrae 10 to 13. Patterson and Kraglievich [15] described the 10th vertebra as a cervical one because it has an *ansa costotransversaria* instead of a floating rib. However, the presence of *processus ventralis corporis* and the differences in the *lacuna interzygapophysialis* between this vertebra and the 9th, point out that the 10th vertebra is the first one of the third region.

The 10th cervical vertebra (Fig. 4) is wider than previous vertebrae in dorsal view. The *processus spinosus* is stout, caudally placed and has a bigger area for the insertion of the *lig. elasticum interlaminare*. There is no *processus caroticus*, but *processus ventralis corporis* is present. The angle measurements (Table 1) indicate a limited dorsal and ventral flexion for the R3 (see next section).

The 11th cervical vertebra is very similar to the 10th. It has a compressed *processus ventralis corporis*, well extended and cranially projected. The *processus spinosus* is big and caudally placed. The insertion area of the *lig. elasticum interlaminare* is wide and creased.

The 12th cervicodorsal vertebra was described by Patterson and Kraglievich [15] as the first cervicodorsal vertebra. This one is stouter than the preceding ones and the main features are the absence of fused ribs (which are free) and the wide development of the *processus spinosus*, with bigger and creased areas for ligament insertion.

The 13th cervicodorsal vertebra is similar, except that the *processus spinosus* is much higher, although it has its distal end broken and the bifurcated condition can not be verified. Ligament insertion areas are higher. The *processus ventralis corporis* is partially eroded and at each side of the corpus the *processi caroticus* can be found.

### Vertebrae with Bifurcated Neural Spines (BNS)

The fifth caudal cervical and first cervicodorsal vertebrae [19] of *Andalgalornis steulleti* have low bifurcated neural spines (metapophyses *sensu* [20]); this means cervical vertebrae 7–9 of R2, 10 and 11 of R3, and the cervicodorsal vertebra 12 of R3. Altogether, 38% of the total cervical vertebrae number of the neck has BNS (Fig. 4). For comparative purposes Table 3 shows the distribution and proportions of vertebrae with BNS in other birds.

Six vertebrae with BNS (vertebrae 11 to 16, being 35% of the total number of vertebrae of the neck) are reported in the Greater Rhea *Rhea americana* [18], [21], [22]. The ibis *Theristicus* Wagler 1832 shows eight vertebrae (vertebra 7th–15th, 47%) with BNS. There are also double spines in the extinct Dromornithidae, Gastornithidae and others Phorusrhacidae [18], [23]. However, BNS are absent in the seriemas and in *Struthio*, one long necked bird that has been traditionally used to be compared with extinct vertebrates (such as non avian dinosaurs) [24].

### Measures, Indices and Angles

Tables 1, 2 and 3 show proportions, measurements and indices taken from the 3rd to the 13th cervical and cervicodorsal vertebrae of *Andalgalornis*. Of the total number of vertebrae of the neck, 40% belongs to the R2 while 30% corresponds to the R3. This arrangement is very similar to those of carnivorous birds like the Hawk *Buteo* and the Barn Owl *Tyto*, but very distinct to the seriema *Chunga* a very striking considering that Cariamidae are the closest extant relatives to the Phorusrhacidae [25], [26]. On the contrary, in the neck of *Rhea americana*, R2 represents the 53% of the total number of the cervical vertebra and flexion between R2 and R3 is located more caudally, between dorsal vertebrae 14 and 15.

Along the neck of *Andalgalornis steulleti,* the corpus length (CL) remains approximately constant recording the lowest values in the 13th vertebra and the highest in the 6th, 7th and 8th vertebrae. The maximum length (ML) noticeably increases in the 5th and 6th vertebrae. However, their value caudally decreases from there. Maximum height (MH) increases from the 6th vertebra, but in the vertebrae 11, 12 and 13 it becomes really high. The anterior width (AW) remains relatively constant with slight increase in the last three vertebrae. Only at the vertebrae 3rd and 4th the anterior width is minor than the caudal width (Cw).

Metric properties of the neck of *Andalgalornis* are also similar to that observed in *Buteo* and *Tyto*. In *Tyto*, in the vertebrae 5 and 6 the maximum length dominates over the anterior width. In *Buteo* instead, the anterior width is larger than the posterior one. The last *Andalgalornis’* vertebrae behave like in *Buteo*: the maximum height is major than the anterior width. None of the measured variables behaves in a similar way to the long necks of *Struthio* (see length dimension, Figure 4 [3]) or *Rhea*. Unexpectedly, vertebrae of the neck of the Black Legged seriema *Chunga* metrically behaves very different from the terror bird *Andalgalornis*. The measured variables in the seriemas however, show a pattern similar to *Rhea* and *Rhynchotus*: the maximum length predominates in all central vertebrae from the fourth to the 12th (from the third to the 13th in *Rhynchotus* and from the fourth to the 15th in *Rhea*). Both in *Rhea* and *Chunga* the cranial width exceeds the value of vertebral body length only in the last vertebrae (the 13th in *Chunga* and the 14th in *Rhea*). The Gull *Larus* (Charadriiformes, Lariidae) shares with *Andalgalornis* the same number of neck vertebrae (13) but the metric behavior of *Larus* is remarkably different from all the species compared: the maximum length is larger in the first nine vertebrae and the cranial width predominates in the remaining ones.

In relation to the angles between vertebrae (Table 1), the only reliable angles values obtained on R1 is between vertebrae 4 and 5 being the ventral angle larger than the dorsal one. The R2 shows significantly low ventral angles values but very high dorsal angles. The dorsal values gradually increase until the articulation between vertebrae 8 and 9. Meanwhile, ventral angles decrease until the articulation of vertebrae 8 and 9. In the R3 the dorsal angles values caudally decrease whereas the ventral angles are variable but low.

## Discussion

Morphologically, it is relatively easy to distinguish which vertebra corresponds to each region of the neck of *Andalgalornis steulleti*. Cervical vertebrae from R1 ventrally have very developed *facies articularis cranialis*, the *arcus vertebrae* is cranially extended as the *corpus*, the *zygapophysis cranialis* are mediocranially sloping and the *lacuna interzygapophysialis* is absent. R2 cervical vertebrae have a low *processus spinosus*, the *concavitas lateralis* are deep, the *lacuna interzygapophysialis* is very well developed adopting a wedge or “V” shape and ventrally, the *corpus* had *processus caroticus*. On the contrary, vertebrae from R3 have a *processus spinosus* high and laterally expanded, the *lacuna interzygapophysialis* is poorly developed and adopts a trapedozoidal shape and the corpus had a *crista ventralis corporis*.

It is clear that the external appearance of the neck does not unequivocally reflect the number of vertebrae that compose it, nor does it reflect folding capability. An externally short neck like in *Buteo* has a high capability of folding in the sagittal plane. Because the possibility of high folding is untenable in the terror bird *Andalgalornis*, it should be considered not a short but a medium-long necked bird.

**Figure 6:**
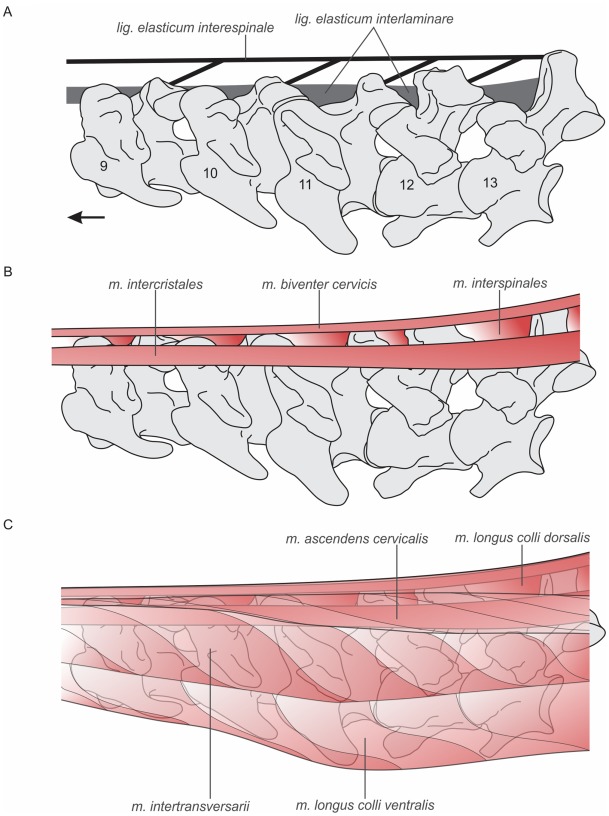
Schematic hypothetical lateral reconstruction of neck muscles and ligaments in *Andalgalornis* based on sites of muscle origin and insertions identified in the holotype and according [**10]**, [22], [30****]. A–C represents successively superficial layers. Arrow indicates cranial direction.

In all birds there is a discontinuous *ligamentum elasticum interlaminare* that connects the *laminae* of adjacent vertebrae [22 and literature cited therein]. This ligament is more developed in the cranial (R1) and caudal (R3) cervical region and helps to keep the head erect when the animal is in neutral position [21]. A second ligament, the *ligamentum elasticum interespinale*, is present only in all the birds that have BNS and occupies the space between both neural spines. Based on a topological criterion, we infer the presence of this ligament in *Andalgalornis* (Fig. 6). This ligament helps to support the weight of the neck when the posterior part descends and falls below the level of origin of the ligament.

Concerning the neck myology (Fig. 6), the *m. biventer cervicis* filled in part the space between the neural spines. Both *m. biventer cervicis* (inserted on the dorsal tubercles or dorsal processes below the *zygapophyses caudalis*) and *m. longus colli dorsalis pars caudalis* are the main epaxial muscles of the neck in all extant birds.

Therefore, the presence of bifurcate neural spines in *Andalgalornis* suggests the presence of a very intricate ligamentary system (with both the *lig. elasticum interspinale* and *ligamentum elasticum interlaminare*) and a very well developed epaxial musculature.

So far, bifurcated neural spines have been reported in birds from different lineages and different trophic habits with large skulls (e.g. *Andalgalornis, Diatryma, Genyornis*) or relatively small size skulls (*Rhea*). Therefore, the presence of BNS in birds is puzzling and warrants further investigation. Outside the clade Aves, the bifurcation of the cervical and dorsal neural spines characterizes several clades of sauropod dinosaurs (e.g., Flagellicaudata), as well as several different genera (*Camarasaurus*, *Opisthocoelicaudia*), all of which evolved independently [22]. The biological significance of the BNS is still unknown but this diversity is an interesting topic to explore phylogenetic constraints and possible adaptive significance of the *lig. elasticum interespinale* [19].

**Figure 7:**
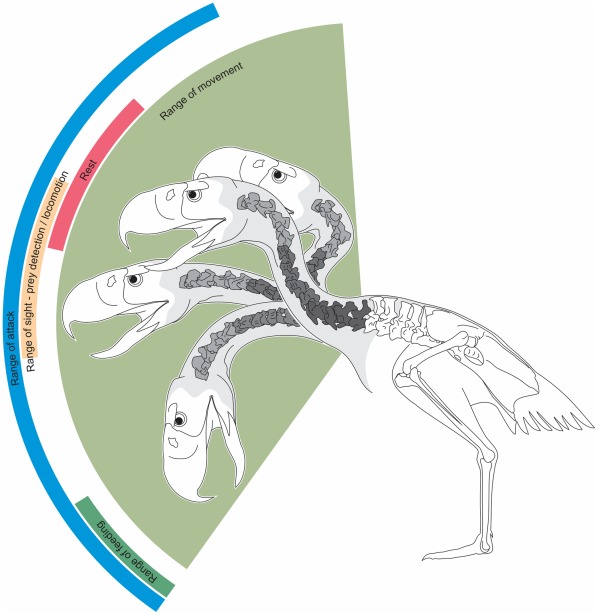
Hypothetical range of dorsoventral movements of the neck of *Andalgalornis* based on [31].

In *Andalgalornis*, the *occiput* and *condylus occipitalis* position indicates that at least the first two cervical vertebrae of R1 could only be located in line with the major axis of the skull. Based on the osteology, other positions would be anatomically and mechanically untenable. R2 and R3 show a marked prevalence of the ventral flexion (high dorsal angles). Also, the maximum potential dorsiflexion of the cervical vertebrae on the sagittal plane (Table 1) suggests that the head could not be located over the back of the body (Fig. 7). In other words, a position of the head over the level of the scapulae exceeds the possible range of motion during life. From the previously mentioned characters as well as the manual manipulation of the cervical vertebrae, it is possible to infer that the neck does not take a complete vertical upright position in a stationary situation (Fig. 7) as in *Rhea* or *Struthio* (see Figure 11 for comparison in [3]).

Throughout the cervical column, the neural spines vary showing the different possibilities of bending of the three sections. If the spine is longer, the lever arm length (the perpendicular distance from the line of action to the pivot) increases magnifying the effective force on the corpus [27]. When the neck is flexed downward, the neural spines transmit tension stresses whereas the vertebral centra resist compression stresses. Because in the cervical column of *Andalgalornis* the neural spines are evident in R1, lower in R2, and conspicuously increases in the R3, it seems possible that during down strokes, R3 concentrates the major stresses.

It is known that the dorsal neck muscles are related with the dissipation of tension stresses during a neutral position of the animal [28]. Judging by the highly developed neck system, it can be supposed that the neck of *Andalgalornis* could be related to two main functions: supporting a particularly big head during normal stance and helping the neck (and the head) to rise after the maximum ventroflexion during a down stroke in the attack of prey. A finite element analysis applied to *Andalgalornis steulleti* [12] suggests that the skull would be prepared to make sudden movements in the sagittal plane to subdue prey. Based on these results, it is interpreted that during an attack phase -downstroke- the head of the animal would quickly move to achieve the maximum ventro-flexion. To start a new phase it is essential that the head and the neck quickly and effectively recover their initial position in height. Piecing altogether, the *lig. elasticum interespinale* and *lig interlaminare* as well as the epaxial muscle (mainly the *m. biventer cervicis*) helped the neck (and the head) to rise after the maximum ventroflexion during a strike.

From the morphology of the vertebrae as a whole and particularly of the transitional vertebrae the cervical flexion patterns can be established. Morphological correlates with activity and capability are difficult to pin down. But combining our results with other evidence is possible to infer some paleobiological aspects of *Andalgalornis*. Our study also indicates which movements are not possible or restricted mainly by mechanical constraints. But those movements that have no restrictions were not necessarily employed by the animal. In other words, our results indicate what kind of movements *Andalgalornis* was able to do (in a vertical plane), but this does not necessary imply that they were done.

The study performed here was uniplane and movements of the neck are multidimensional by nature. Future studies should consider all possibilities of the behavior of *Andalgalornis*. Also, this study is the first one to analyze the potential functioning of a phorusrhacids neck in its entirety. In a broad sense, we may assume that the necks of other large phorusrhacids (big headed birds with high and compressed beaks) would respond similarly to that of *Andalgalornis*. We are now one step closer to understanding the phorusrhacids lifestyle.

## Materials and Methods

Cervical vertebrae of *Andalgalornis steulleti* were studied at División Paleontología Vertebrados of Museo de La Plata (cast MLP 604-I) and Field Museum of Natural History (FM-P14357). The atlas is not preserved (the atlas available in some collections or exhibitions is a model based on *Mesembriornis milneedwardsi*). The axis is highly distorted and eleven articulated cervical vertebrae are available to study. The cervical vertebrae of *Procariama simplex* FM-P14525, *Mesembriornis milneedwardsi* MACN 5944 and *Psilopterus lemoinei* AMNH 9157, AMNH 9257 and YPM-PU15402 were studied for comparison. Nine modern species representing a wide range of external neck length were evaluated: long necks *Cygnus melancorhyphus* MLP 688, *Nycticorax nycticorax* MLP 69, *Theristicus caudatus* MLP 474, and *Rhea americana* MLP 650; medium neck length *Rhynchotus rufescens* MLP 30 and *Larus* sp. MLP 577, short necks *Buteo magnirostris* MLP 362, *Tyto alba* MLP 53 and, *Ramphastos toco* MLP 314 (Fig. 2). Also, the medium neck length Cariamidae *Chunga burmeisteri* MACN 2351a, MLP 52 was included in the analysis because of its close phylogenetic relationships with phorusrhacids. The osteological samples for comparison studied come from the Museo de La Plata (MLP) and Museo de Ciencias Naturales “Bernardino Rivadavia” (MACN).

Descriptions and observations are organized around the three regions proposed by Boas [6]. Two transitional vertebrae (TV) separated region 1 (R1) from region 2 (R2) and region 2 from region 3 (R3). Studying penguins Guinard et al. [9] applied an alternative approach identifying biomechanical zones within the cervical system that they call “modules” [following 29]. In the same paper they propose that their method can be applied to any other avian taxa [9]. However, we prefer to apply here the classical proposal of Boas [6] because regions are easy to delimit and to compare between the set of birds chosen here.

The anatomical terminology is given in the text with their Latin denomination (as used by Baumel and Witmer [10]) and English equivalents.

Ten biometric measurements were taken directly from the material using a digital caliper (Fig. 3). Each measurement was taken three times, averaged and the standard error was calculated. When the error was greater than 0.5 the three measurements were repeated. Two indices were calculated: the development and cranial projection of *facies articularis cranialis* and the elongation index, the latter in the same way as Guinard et al. [9]. The former gives an idea of the dorsal folding of the neck, e.g. vertebrae with low values have been weakly folded and the latter is an expression of the cohesion of the neck, e.g. regions formed by vertebrae with low values will be more cohesive.

We applied the zygapophyseal alignment model [24] to establish the possible range of movement of the neck of *Andalgalornis*. The cervical vertebrae were ‘articulated’ and manually manipulated on a flat horizontal surface parallel to the sagittal plane, to recreate the range of allowable dorsoventral flexion. Therefore, for the purpose of modeling flexion and motion we reduced the head-neck complex to a planar multi-element system. The intervertebral space was estimated as 1 mm and the minimum functional overlap of the zygapophysis as 3 mm. However, there is no direct way of knowing how much cartilage would originally have been present in a neck of a phorusrhacid. In this sense, our reconstructions of possible neck poses are conservative. Photographs were taken showing the maximum neck flexion capacity between regions, concentrating on the bending at the level of transitional vertebrae. We measured with a goniometer the dorsal and ventral angles between one vertebra and the next one when vertebrae of the neck are assembled directly on the material. During the dorsal flexion (caudal loop or kyphotic), dorsal angles exhibit low values. On the contrary, during ventral flexion (rostral loop or lordotic), the ventral angles show low values.

The material was photographed using a reflex photographic camera. The images were processed with Adobe PhotoShop CS3 software to produce the final figures.

Institutional Abbreviations: MLP Museo de La Plata, AMNH American Museum of Natural History (New York City), FMNH Field Museum of Natural History (Chicago), YPM-PU Yale Peabody Museum (New Haven).
